# Targeted proteomic analysis dataset of archival core human kidney biopsies to investigate the biology of hypertensive nephropathy^✰^

**DOI:** 10.1016/j.dib.2022.107805

**Published:** 2022-01-06

**Authors:** Georgios Barkas, Manousos Makridakis, Hariklia Gakiopoulou, Demetrios Vlahakos

**Affiliations:** a Biomedical Research Foundation of the Academy of Athens, Greece; b National and Kapodistrian University of Athens Medical School, Athens, Greece

**Keywords:** Parallel Reaction Monitoring (PRM), Proteomics, Immunofluorescence, Hypertensive nephropathy, Human Kidney Biopsies, SGLT2, CLIC4, Proximal tubules

## Abstract

Hypertensive nephropathy is the second most frequent cause of end-stage renal disease in western societies. In previous experiments in our laboratory with proteomic analysis of renal parenchyma of SHR hypertensive animals, we identified two molecules, namely SGLT2 and CLIC4, associated with the development of hypertension. Here, we apply the methodology of targeted proteomic analysis in kidney biopsies from patients with hypertensive nephropathy to study the role of SGLT2 and CLIC4 in the pathogenesis of the disease. Relative quantification data of SGLT2 and CLIC4 via means of targeted proteomic analysis in human kidney biopsies from hypertensive patients and normotensive controls are reported. In addition, validation data of the proteomic results via immunofluorescence are presented. Renal tissue biopsies (*N* = 17) from archival material of patients with hypertensive nephropathy and normotensive controls were used. Targeted proteomic analysis was performed using the method: ``Parallel Reaction Monitoring'' (PRM) in renal parenchyma of hypertensive and normotensive patients for the selective identification of SGLT2 and CLIC4 and the relative quantification of their expression using proteotypic peptides for each protein. The expression of SGLT2 molecule was also confirmed by immunofluorescence followed by quantification of fluorescence intensity. According to PRM, the SGLT2 protein was found with reduced and the CLIC4 protein with increased expression levels in hypertensive patients compared to normotensive controls. Comparison of representative immunofluorescence images confirmed a decrease in the expression of SGLT2 in the brush border of proximal tubular epithelial cells in hypertensive patients. Our data show changes in the tubular compartment of the kidney and especially in the proximal tubules associated with the hypertensive nephropathy. The clinical significance of these findings should be further explored for the discovery and/or confirmation of novel therapeutic approaches and biomarkers in the development of hypertensive kidney disease.

**Specifications Table**

**Table:** 

Subject	Biology
Specific subject area	Proteomics
Type of data	Raw data, Tables and figures
How data was acquired	Q Exactive high-resolution mass spectrometer (Thermo Fisher Scientific, USA) coupled with an Ultimate 3000 Nano-flow HPLC system (Thermo Fisher Scientific, USA) was used to acquire PRM data.
Data format	Raw and analyzed data
Parameters for data collection	LC-MS/MS spectra were collected from 8 biological replicates of normotensive and 5 biological replicates from hypertensive samples.
Description of data collection	• Archival core human kidney biopsies. • Protein extraction from FFPE sections • PRM analysis • Data processing
Data source location	Biomedical Research Foundation, Academy of Athens, Athens, Greece
Data accessibility	Raw data are available via Mendeley Data https://data.mendeley.com/datasets/jv4ht2rvhx/2

**Value of the Data**

• This dataset includes novel research findings regarding the expression of two molecules, SGLT2 and CLIC4, in the proximal tubules of hypertensive and normotensive patients.
• The data presented provide evidence that proteomic changes occur in the tubular compartment of human kidney due to hypertension.
• These data may be of interest to the researchers interested in the development of hypertensive nephropathy in humans.
• The protocol applied for the protein extraction from FFPE archival core biopsies and the subsequent proteomics analysis could be useful for researchers that have access to this type of material instead of fresh frozen tissue samples.

## 1 Data Description

**Fig. 1:**
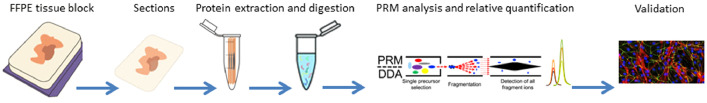
Study workflow: Archival core biopsies were sectioned to 5 µm sections. After deparaffinization and rehydration of the sections, proteins were extracted and digested with trypsin. PRM analysis and relative quantification of SGLT2 and CLIC4 was performed followed by immunofluorescence validation of SGLT2.

**Fig. 2:**
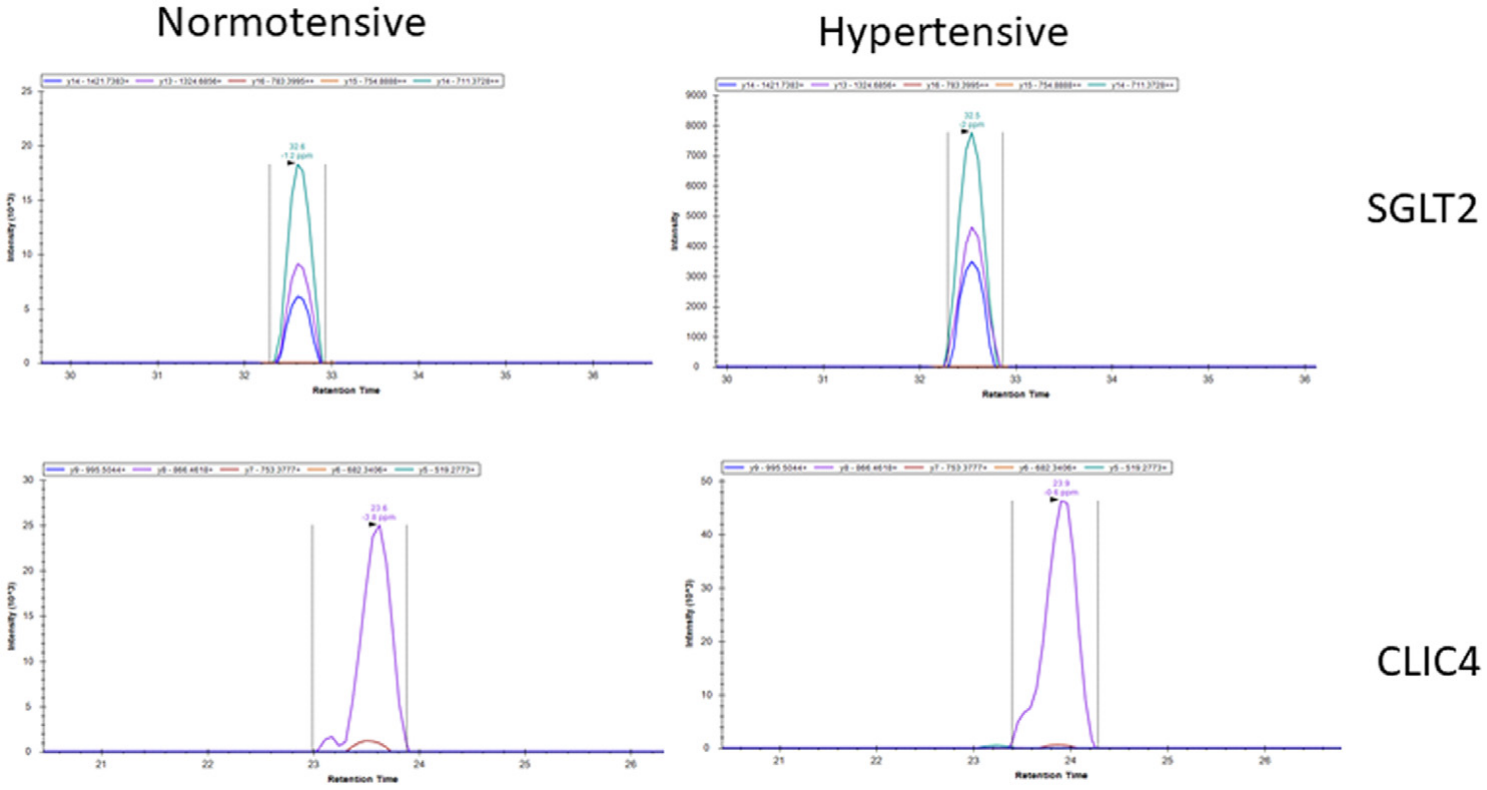
Representative images of the PRM analysis for SGLT2 (upper panel) and CLIC4 (lower panel) in normotensive and hypertensive clinical samples. SGLT2 was up-regulated in normotensive samples whereas CLIC4 had higher expression in hypertensive samples.

The dataset describes results obtained from the PRM analysis in archival core biopsies of hypertensive (*N* = 5) and normotensive (*N* = 8) individuals for the selective identification and relative quantification of SGLT2 and CLIC4 proteins. The workflow applied for sample collection, protein extraction, targeted proteomics analysis (PRM) and immunofluorescence validation is shown in Fig. 1 and detailed in the experimental design, materials, and methods section. PRM data were analyzed with Skyline software. Selective identification and relative quantification of SGLT2 and CLIC4 were performed based on proteotypic peptides. SGLT2 was found to be down-regulated (Table 1) whereas CLIC4 was up-regulated (Table 2) in hypertensive patients compared to normotensive controls. Representative images of the spectra obtained from the PRM analysis for SGLT2 and CLIC4 are shown in Fig. 2. Down-regulation of SGLT2 and up-regulation of CLIC4 in hypertensive patients compared to normotensive controls was observed (Fig. 2).

**Table 1:** PRM data analysis for SGLT2 based on the proteotypic peptide: “GGVGSPPPLTQEEAAAAAR”. Total area of the precursor ion fragments was utilized for the relative quantification of SGLT2 in normotensive and hypertensive samples.

Peptide Sequence	Protein Entry Name	Replicate Name	Transitions (Precursor Ion/Fragment Ions)	Total Area
GGVGSPPPLTQEEAAAAAR	SC5A2_HUMAN	Normotensive 1	889.9552++/1421.7383+ (y14), 1324.6856+ (y13), 783.3995++ (y16), 754.8888++ (y15), 711.3728++ (y14)	572,734
GGVGSPPPLTQEEAAAAAR	SC5A2_HUMAN	Normotensive 2	889.9552++/1421.7383+ (y14), 1324.6856+ (y13), 783.3995++ (y16), 754.8888++ (y15), 711.3728++ (y14)	445,142
GGVGSPPPLTQEEAAAAAR	SC5A2_HUMAN	Normotensive 3	889.9552++/1421.7383+ (y14), 1324.6856+ (y13), 783.3995++ (y16), 754.8888++ (y15), 711.3728++ (y14)	1,068,914
GGVGSPPPLTQEEAAAAAR	SC5A2_HUMAN	Normotensive 4	889.9552++/1421.7383+ (y14), 1324.6856+ (y13), 783.3995++ (y16), 754.8888++ (y15), 711.3728++ (y14)	238,409
GGVGSPPPLTQEEAAAAAR	SC5A2_HUMAN	Normotensive 5	889.9552++/1421.7383+ (y14), 1324.6856+ (y13), 783.3995++ (y16), 754.8888++ (y15), 711.3728++ (y14)	162,172
GGVGSPPPLTQEEAAAAAR	SC5A2_HUMAN	Normotensive 6	889.9552++/1421.7383+ (y14), 1324.6856+ (y13), 783.3995++ (y16), 754.8888++ (y15), 711.3728++ (y14)	132,998
GGVGSPPPLTQEEAAAAAR	SC5A2_HUMAN	Normotensive 7	889.9552++/1421.7383+ (y14), 1324.6856+ (y13), 783.3995++ (y16), 754.8888++ (y15), 711.3728++ (y14)	244,450
GGVGSPPPLTQEEAAAAAR	SC5A2_HUMAN	Normotensive 8	889.9552++/1421.7383+ (y14), 1324.6856+ (y13), 783.3995++ (y16), 754.8888++ (y15), 711.3728++ (y14)	62,743

GGVGSPPPLTQEEAAAAAR	SC5A2_HUMAN	Hypertensive 1	889.9552++/1421.7383+ (y14), 1324.6856+ (y13), 783.3995++ (y16), 754.8888++ (y15), 711.3728++ (y14)	270,134
GGVGSPPPLTQEEAAAAAR	SC5A2_HUMAN	Hypertensive 2	889.9552++/1421.7383+ (y14), 1324.6856+ (y13), 783.3995++ (y16), 754.8888++ (y15), 711.3728++ (y14)	0
GGVGSPPPLTQEEAAAAAR	SC5A2_HUMAN	Hypertensive 3	889.9552++/1421.7383+ (y14), 1324.6856+ (y13), 783.3995++ (y16), 754.8888++ (y15), 711.3728++ (y14)	48,379
GGVGSPPPLTQEEAAAAAR	SC5A2_HUMAN	Hypertensive 4	889.9552++/1421.7383+ (y14), 1324.6856+ (y13), 783.3995++ (y16), 754.8888++ (y15), 711.3728++ (y14)	76,062
GGVGSPPPLTQEEAAAAAR	SC5A2_HUMAN	Hypertensive 5	889.9552++/1421.7383+ (y14), 1324.6856+ (y13), 783.3995++ (y16), 754.8888++ (y15), 711.3728++ (y14)	37,137

**Table 2:** PRM data analysis for CLIC4 based on the proteotypic peptide: “EVEIAYSDVAK”. Total area of the precursor ion fragments was utilized for the relative quantification of CLIC4 in normotensive and hypertensive samples.

Peptide Sequence	Protein Entry Name	Replicate Name	Transitions (Precursor Ion/Fragment Ions)	Total Area
EVEIAYSDVAK	CLIC4_HUMAN	Normotensive 1	612.3113++/995.5044+ (y9), 866.4618+ (y8), 753.3777+ (y7), 682.3406+ (y6), 519.2773+ (y5)	463,469
EVEIAYSDVAK	CLIC4_HUMAN	Normotensive 2	612.3113++/995.5044+ (y9), 866.4618+ (y8), 753.3777+ (y7), 682.3406+ (y6), 519.2773+ (y5)	1,085,482
EVEIAYSDVAK	CLIC4_HUMAN	Normotensive 3	612.3113++/995.5044+ (y9), 866.4618+ (y8), 753.3777+ (y7), 682.3406+ (y6), 519.2773+ (y5)	702,872
EVEIAYSDVAK	CLIC4_HUMAN	Normotensive 4	612.3113++/995.5044+ (y9), 866.4618+ (y8), 753.3777+ (y7), 682.3406+ (y6), 519.2773+ (y5)	819,764
EVEIAYSDVAK	CLIC4_HUMAN	Normotensive 5	612.3113++/995.5044+ (y9), 866.4618+ (y8), 753.3777+ (y7), 682.3406+ (y6), 519.2773+ (y5)	324,388
EVEIAYSDVAK	CLIC4_HUMAN	Normotensive 6	612.3113++/995.5044+ (y9), 866.4618+ (y8), 753.3777+ (y7), 682.3406+ (y6), 519.2773+ (y5)	543,381
EVEIAYSDVAK	CLIC4_HUMAN	Normotensive 7	612.3113++/995.5044+ (y9), 866.4618+ (y8), 753.3777+ (y7), 682.3406+ (y6), 519.2773+ (y5)	501,442
EVEIAYSDVAK	CLIC4_HUMAN	Normotensive 8	612.3113++/995.5044+ (y9), 866.4618+ (y8), 753.3777+ (y7), 682.3406+ (y6), 519.2773+ (y5)	539,180
EVEIAYSDVAK	CLIC4_HUMAN	Hypertensive 1	612.3113++/995.5044+ (y9), 866.4618+ (y8), 753.3777+ (y7), 682.3406+ (y6), 519.2773+ (y5)	1,065,856
EVEIAYSDVAK	CLIC4_HUMAN	Hypertensive 2	612.3113++/995.5044+ (y9), 866.4618+ (y8), 753.3777+ (y7), 682.3406+ (y6), 519.2773+ (y5)	377,301
EVEIAYSDVAK	CLIC4_HUMAN	Hypertensive 3	612.3113++/995.5044+ (y9), 866.4618+ (y8), 753.3777+ (y7), 682.3406+ (y6), 519.2773+ (y5)	634,829
EVEIAYSDVAK	CLIC4_HUMAN	Hypertensive 4	612.3113++/995.5044+ (y9), 866.4618+ (y8), 753.3777+ (y7), 682.3406+ (y6), 519.2773+ (y5)	3,398,850
EVEIAYSDVAK	CLIC4_HUMAN	Hypertensive 5	612.3113++/995.5044+ (y9), 866.4618+ (y8), 753.3777+ (y7), 682.3406+ (y6), 519.2773+ (y5)	3,312,541

**Fig. 3:**
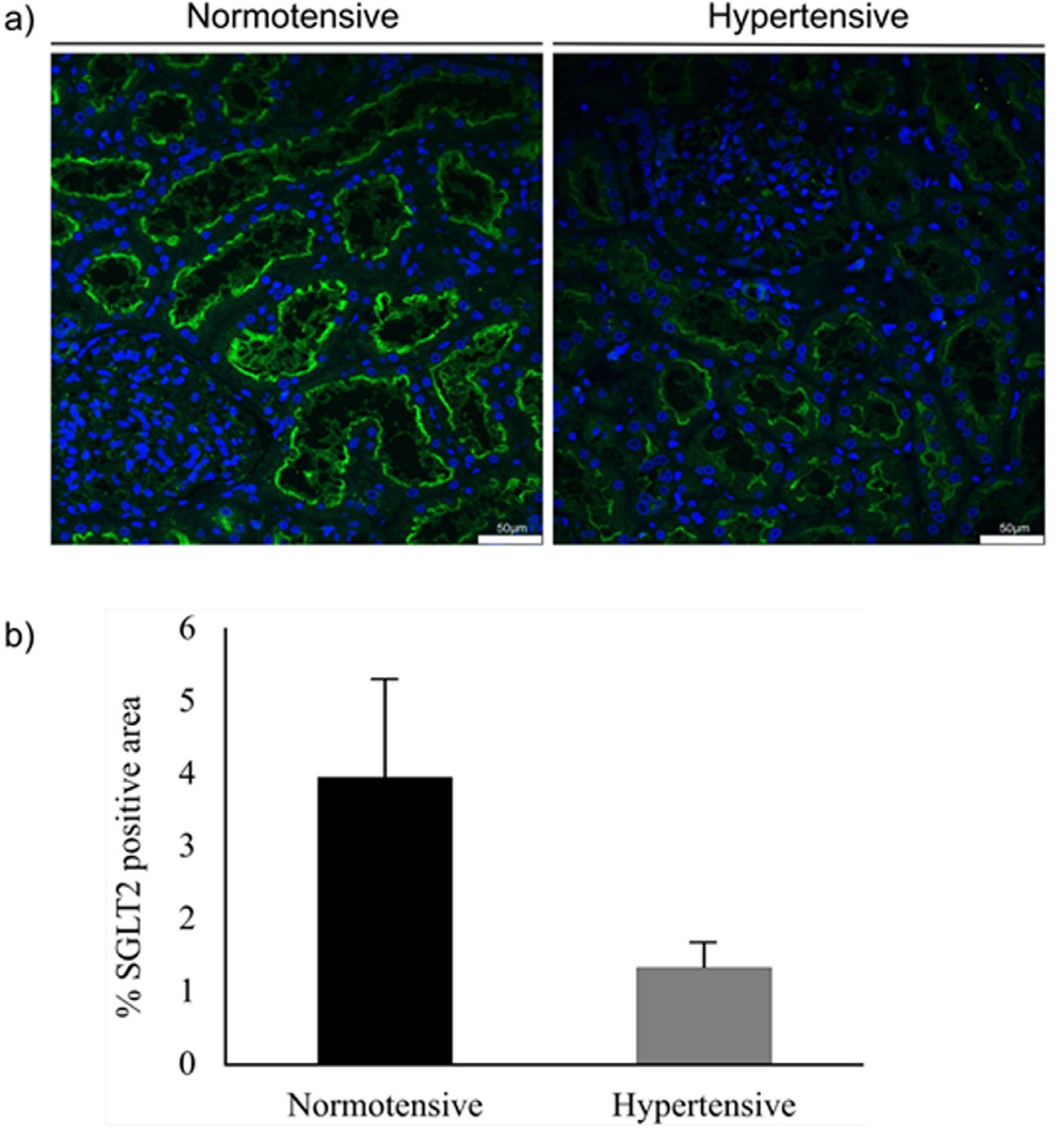
Immunofluorescence validation of SGLT2. (a) Representative IF images of normotensive and hypertensive kidney sections with anti-SGLT2 antibody show significant downregulation of SGLT2 in hypertensive patients compared to normotensive controls. Scale bar: 50 µm. (b) Quantification of SGLT2 staining in non-overlapping images shows significantly reduced SGLT2-positive area in hypertensive samples.

Proteomics dataset was validated by immunofluorescence of the SGLT2 protein (Fig. 3). Archival core biopsies (different from the ones used for the targeted proteomics analysis) of hypertensive (*N* = 4) were used for the validation. As control were used kidney sections from the same archival core biopsies of normotensive individuals (*N* = 8) used for the PRM analysis. Quantification of the fluorescence intensity showed a decrease in SGLT2 expression in hypertensive patients compared to normotensive controls (Fig. 3).

All the raw files from PRM analysis of SGLT2 and CLIC4 as well as the immunofluorescence images from the SGLT2 validation are available via Mendeley Data repository [1].

## 2 Experimental Design, Materials and Methods

### 2.1 Archival core human kidney biopsies

The biopsies were collected at Laikon University Hospital, Athens. Sections of 5 µm thick FFPE tissue per sample were utilized for the targeted proteomics analysis and the immunofluorescence validation.

### 2.2 Protein extraction from archival FFPE core human kidney biopsies

Protein extraction from FFPE tissue samples was performed as previously described [2]. Briefly, FFPE samples were deparafinized in xylene (2 incubations for 5 min followed by a final one for 1 min). The sections were then rehydrated with a series of washes with decreasing ethanol concentration (100% ethanol for 2 min, 95% ethanol for 1 min, 70% ethanol for 1 min) followed by a final wash with ultra-pure water for 1 min. After rehydration, samples were air-dried for 30 min at RT. Dried samples were resuspended in lysis buffer consisting of 100 mM Tris-HCl pH 7.6, 4% SDS, 100 mM DTE. Homogenization was performed with a bead homogenizer (Bullet Blender Storm BBY24M, Next Advance, USA) by utilizing 0.9–2.0 mm stainless steel beads. In addition, samples were sonicated for 3 cycles of 5 s each with a tip sonicator (36% power used) and then incubated for 1 h at 90 °C. Samples were then centrifuged for 10 min at 14,000 × g, RT and the supernatants (total protein extracts) were kept in clean tubes. Protease inhibitors were added to the extracts which were stored at −80 °C until use.

### 2.3 Sample preparation for the proteomics analysis

Samples were prepared with the GeLC-MS method as previously described [3]. Protein extracts were concentrated with Amicon Ultra Centrifugal Filters (3 kDa MW cutoff) and subjected to buffer exchange with 50 mM ammonium bicarbonate, pH 8.5 to a final volume of 20 µL. Samples were loaded on the polyacrylamide gel and the GeLC-MS protocol was applied. In brief, electrophoresis was stopped when the samples just entered the separating gel. The gel was fixed with 30% methanol, 10% acetic acid for 30 min and then washed with water (3 × 5 min washes) before stained o/n with coomassie colloidal blue. Excess of stain was washed with water and the protein bands were excised from the gel and sliced into small pieces. Gel pieces were destained with 40% acetonitrile, 50 mM ammonium bicarbonate (destain solution) followed by reduction with 10 mM DTE in 100 mM ammonium bicarbonate for 20 min at RT. After reduction, samples were alkylated with 54 mM iodoacetamide in 100 mM ammonium bicarbonate for 20 min in the dark at RT. A series of washes was performed starting with 100 mM ammonium bicarbonate for 20 min, RT, followed by a wash with destain solution and a final wash with ultra-pure water under the same conditions. Gel pieces were dried in a centrifugal vacuum concentrator and trypsinization was performed by adding 600 ng trypsin per sample (trypsin stock solution: 10 ng/µL in 10 mM ammonium bicarbonate pH 8.5) for 12–16 h, at RT in the dark under humidified conditions. Tryptic peptides were extracted by incubation with 50 mM ammonium bicarbonate for 15 min, RT followed by incubation with 5% formic acid, 50% acetonitrile (15 min, RT) which was performed twice. The extracted peptides were dried with a centrifugal vacuum concentrator and stored at −80 °C until use.

### 2.4 Proteotypic peptide selection for PRM analysis

Skyline software indicated proteotypic peptides (peptides uniquely representing the target protein) after importing a spectral library from the National Institute of Standards and Technology (NIST) downloaded from http://www.nist.gov/. In addition, proteotypic peptides were evaluated using the Protein Basic Local Alignment Search Tool (BLAST, http://blast.ncbi.nlm.nih.gov) to ensure their proteotypicity. The proteotypic peptide with the best quality spectrum for SGLT2 and CLIC4 was selected for relative quantification in the clinical samples. The information with regards to the transitions (precursor ion → fragment ion) selected for each proteotypic peptide are included in Tables 1 and 2. Briefly, for the SGLT2 proteotypic peptide (GGVGSPPPLTQEEAAAAAR) the following transitions were selected:

(1) precursor ion: 889.9552++, fragment ion: 1421.7383+ (y14)
(2) precursor ion: 889.9552++, fragment ion: 1324.6856+ (y13)
(3) precursor ion: 889.9552++, fragment ion: 783.3995++ (y16)
(4) precursor ion: 889.9552++, fragment ion: 754.8888++ (y15)
(5) precursor ion: 889.9552++, fragment ion: 711.3728++ (y14)

whereas for the CLIC4 proteotypic peptide (EVEIAYSDVAK) the following transitions were selected:

(1) precursor ion: 612.3113++, fragment ion: 995.5044+ (y9)
(2) precursor ion: 612.3113++, fragment ion: 866.4618+ (y8)
(3) precursor ion: 612.3113++, fragment ion: 753.3777+ (y7)
(4) precursor ion: 612.3113++, fragment ion: 682.3406+ (y6)
(5) precursor ion: 612.3113++, fragment ion: 519.2773+ (y5)

### 2.5 PRM

The extracted peptides were resuspended in 10 µL mobile phase A (0.1% formic acid, pH 3.5) and the PRM analysis was performed with an UltiMate 3000 Nano HPLC Dionex Ultimate® 3000 RSLS system (Dionex™; Thermo Fisher Scientific, Inc.) coupled to a Q Exactive (Thermo Fisher Scientific, Inc) mass spectrometer operating in PRM mode as previously described [4]. A volume of 5 µL was injected into the chromatography system and peptides were separated in a C18 column (75 µm × 50 cm, 2 µm, 100 Å) during a 70 min run (0.3 µL/min flow rate) using a gradient of up to 80% mobile phase B (0.1% formic acid in acetonitrile) for elution. The column was washed and re-equilibrated prior to each sample injection. The eluent was ionized using a Proxeon nano spray ESI source operating in positive ion mode. Gaseous phase transition of the separated peptides was achieved with positive ion electrospray ionization applying a voltage of 2.5 kV. Resolution was 70,000 for the MS and 35,000 for the MS2.

### 2.6 Data analysis and processing

The PRM data files were analyzed with the Skyline software. All chromatograms were manually inspected to ensure the good quality and accurate peak picking. The peak areas of the transitions (fragment ions) of the hypertensive samples was compared with the peak areas of the normotensive controls and the ratio obtained was used for the relative quantification of SGLT2 and CLIC4 in the clinical samples (Fig. 2 and Tables 1 and 2).

### 2.7 Validation of PRM by immunofluorescence

FFPE kidney sections from hypertensive and normotensive patients were rehydrated, treated for antigen retrieval with 10 mmol/L sodium citrate (pH 6.0) at 80 °C for 30 min, blocked with a 5% FBS 0.1% Triton solution, incubated with primary antibody to SGLT2 (ab85626, Abcam) at 1:50 dilution overnight at 4 °C, developed using Alexa Fluor fluorescent secondary antibody (Molecular Probes), counterstained with DAPI and mounted in Mowiol 4-88 (Calbiochem). As a control, sections were also incubated with either only the secondary antibody or non-specific IgG in a similar concentration with that of anti-SGLT2 antibody. In randomly selected immunofluorescence images from hypertensive and normotensive patients, the SGLT2 positive area (expressed as percentage of total area) was quantified using software ImageJ (https://imagej.nih.gov/ij/index.html). Results are presented as means ± SD (Fig. 3).

## Ethics Statement

The study was conducted in accordance with the World Medical Association Declaration of Helsinki. All the samples were processed and used in line to ethics requirements. The study was approved by the bioethics committee of the Medical School of Athens (reference number 38, 19/11/2018).

## CRediT authorship contribution statement

**Georgios Barkas:** Conceptualization, Investigation, Validation, Writing – original draft. **Manousos Makridakis:** Conceptualization, Investigation, Data curation, Methodology, Writing – original draft. **Hariklia Gakiopoulou:** Supervision, Writing – review & editing. **Demetrios Vlahakos:** Supervision, Writing – review & editing.

## Declaration of Competing Interest

The authors declare that they have no known competing financial interests or personal relationships, which have or could be perceived to have influenced the work reported in this article.
